# A review of embodied intelligence systems: a three-layer framework integrating multimodal perception, world modeling, and structured strategies

**DOI:** 10.3389/frobt.2025.1668910

**Published:** 2025-11-06

**Authors:** Yunwei Zhang, Jing Tian, Qiaochu Xiong

**Affiliations:** 1 Faculty of Information Engineering and Automation, Kunming University of Science and Technology, Kunming, China; 2 Higher Educational Key Laboratory for Industrial Intelligence and Systems of Yunnan Province, Kunming, China

**Keywords:** embodied AI, multimodal learning, world models, cross-modal learning, reinforcement learning, sim-to-real transfer

## Abstract

Embodied intelligent systems build upon the foundations of behavioral robotics and classical cognitive architectures. They integrate multimodal perception, world modeling, and adaptive control to support closed-loop interaction in dynamic and uncertain environments. Recent breakthroughs in Multimodal Large Models (MLMs) and World Models (WMs) are profoundly transforming this field, providing the tools to achieve its long-envisioned capabilities of semantic understanding and robust generalization. Targeting the central challenge of how modern MLMs and WMs jointly advance embodied intelligence, this review provides a comprehensive overview across key dimensions, including multimodal perception, cross-modal alignment, adaptive decision-making, and Sim-to-Real transfer. Furthermore, we systematize these components into a three-stage theoretical framework termed “Dynamic Perception–Task Adaptation (DP-TA)”. This framework integrates multimodal perception modeling, causally driven world state prediction, and semantically guided strategy optimization, establishing a comprehensive “perception–modeling–decision” loop. To support this, we introduce a “Feature-Conditioned Modal Alignment (F-CMA)” mechanism to enhance cross-modal fusion under task constraints.

## 1 Introduction

The early paradigm of artificial intelligence was largely grounded in the concept of “Disembodied intelligence”. The central goal of this approach was to mimic human intelligent behavior by focusing on enabling machines to simulate human-like thought processes. However, this was done without considering a physical body or an environmental context. These systems’ reasoning relied on static data and pre-defined rules. Because they lacked direct perception and interaction with the physical world, they faced significant limitations, often showing poor generalization, weak adaptability to new tasks, and inefficient interactions in real-world scenarios (Nathan, 2023). Furthermore, real-world environments are increasingly characterized by uncertainty, sensory complexity, and task diversity, making the limitations of traditional disembodied AI increasingly clear. This has created a pressing need to develop intelligent systems that can interact effectively with their environment and adapt their structure accordingly.

**FIGURE 1:**
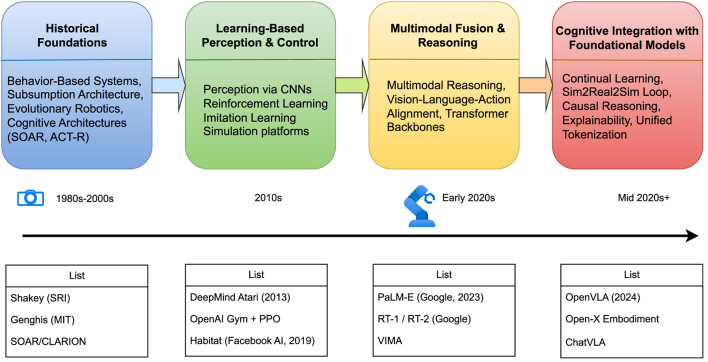
Development timeline of embodied intelligence.

In sharp contrast, the embodied intelligence paradigm emphasizes that intelligence arises through continuous closed-loop interaction between an agent and its environment. Such agents are defined by their physical bodies, sensors, and effectors, which together ground perception and action. The idea is not entirely new; its philosophical and engineering roots can be traced back to the cybernetics movement. Subsequent advances in behavioral robotics (Brooks, 1999; Arkin, 1998) and cognitive architecture theories (Laird et al., 1987) laid the foundations for the principles of embodiment and the perception–decision–execution loop. Building on these ideas, researchers demonstrated how autonomous agents could exhibit robust and adaptive behaviors in complex environments (Pfeifer and Scheier, 2001). This trajectory was further advanced by pioneering work in Evolutionary Robotics (Cliff et al., 1993), which explored automatic controller design, and by cognitive architectures like CLARION (Sun, 2007) and the Multilevel Darwinist Brain (MDB) (Bellas et al., 2010), which integrated learning, reasoning, and embodiment long before the current era of large models. The historical trajectory of embodied intelligence is shown in Figure 1.

For many years, applying these principles in practice has been challenging due to limitations in perception, computation, and reasoning. Recently, however, advances in multimodal large models (MLMs) and world models (WMs) have opened new possibilities. These developments provide researchers with powerful tools to tackle longstanding challenges, bringing us closer to building the embodied agents envisioned in earlier research. Driven by these technologies, the field is undergoing a profound transformation from early modular integration architectures to unified modeling frameworks (Roy et al., 2021). MLMs, including RT-2 (Zitkovich et al., 2023) and OpenVLA (Kim et al., 2024), are built on cross-modal Transformer architectures. This design enables them to create a unified representation from different input types, such as vision and language, and perform joint reasoning to achieve end-to-end control that directly translates natural language commands into physical actions. Concurrently, WMs (Ha and Schmidhuber, 2018b) aim to build an internal understanding of an environment by learning its latent states, enabling them to simulate potential future states and model causal relationships. This capability provides agents with a deeper understanding and facilitates effective knowledge transfer across different tasks (Deitke et al., 2020).

The combination of MLMs and WMs brings notable benefits, including improved system generality, support for cross-task learning, and facilitated deployment from simulation to reality (Sim-to-Real). This paper reviews how these modern advancements are revolutionizing the implementation of embodied systems. Our analysis centers on the synergistic integration of MLMs and WMs as a central focus for advancing embodied intelligence. The discussion is organized around key dimensions such as multimodal perception, cross-modal alignment, and Sim-to-Real transfer. Despite these advances, important gaps remain. Specifically, there is no complete framework that integrates all components systematically. As a result, issues such as information coupling, module decoupling, and interface design are still unresolved (Liu et al., 2025).

**FIGURE 2:**
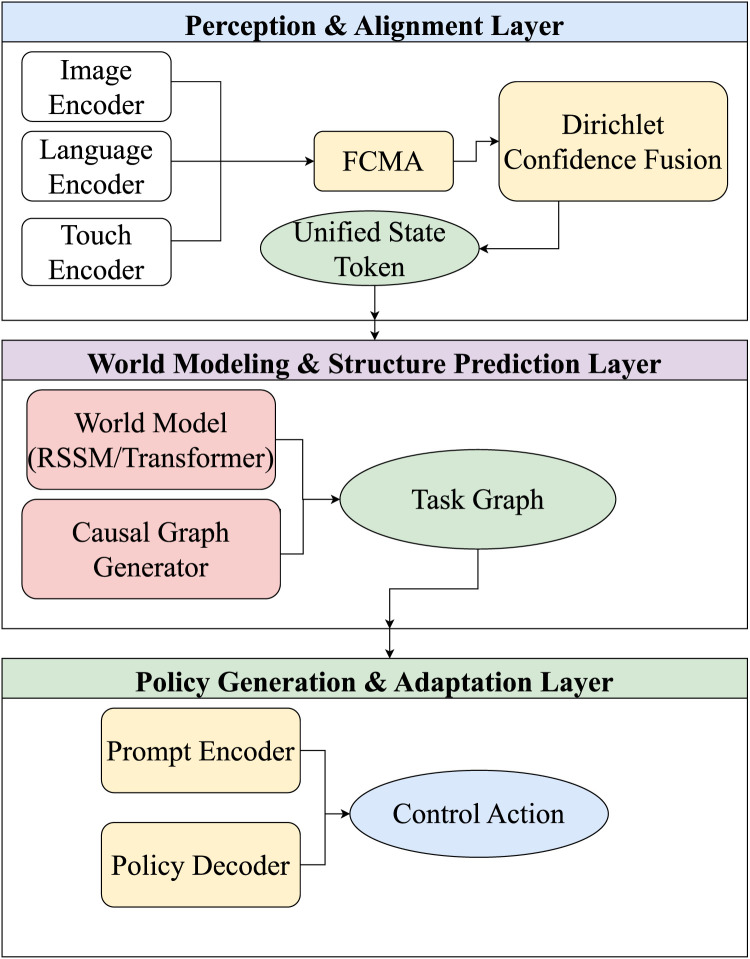
Dynamic perception–task adaptation framework.

To address these challenges, this review proposes a structured modeling framework for embodied intelligent systems: the DP-TA (Dynamic Perception–Task Adaptation) three-layer fusion architecture. This framework decomposes embodied systems into three core functional layers: multimodal perception and alignment, world modeling and task graph generation, and policy adaptation and execution scheduling. These layers correspond to the key processing stages from perceptual input to action output, as illustrated in Figure 2.

To support this architecture, we introduce the Feature-Conditioned Modal Alignment (F-CMA) mechanism, which models how task semantics guide perceptual fusion strategies, thereby enhancing the system’s goal consistency and adaptability. The DP-TA framework not only provides a unified interface specification for multimodal perception, large-scale cognitive modeling, and policy execution but also offers a structured methodological framework for constructing embodied systems with high task generalization and flexible deployment capabilities.

The core contributions of this study are summarized as follows:

1. We systematize the structural fusion of embodied intelligent systems into a framework termed DP-TA, which clearly delineates the functional boundaries of the three core layers—perception, modeling, and policy control—and their collaborative coupling paths.
2. We introduce the F-CMA mechanism, enriching the methodology for cross-modal information alignment.
3. From a system-functional perspective, we synthesize representative mechanisms and synergistic structures that integrate multimodal large models and world models, analyzing their roles in behavior generation and task planning.
4. We examine core mechanisms and optimization pathways for the policy layer, encompassing advanced approaches such as Prompt-Policy structures, tokenized state representations, and skill graph scheduling. We also systematically identify key system-level optimization strategies for task deployment and module sharing.
5. We identify core challenges in current research and outline future trends, focusing on semantic consistency modeling, multi-policy architecture fusion, lightweight world model construction, and multi-task adaptation.

## 2 Multimodal perception and dynamic alignment

### 2.1 Research background and system value of multimodal perception

The perception module has long been recognized as the critical starting point for environmental information flow in intelligent systems, with its performance fundamentally determining the quality of subsequent modeling and decision-making processes. While early embodied systems relied on traditional sensor fusion techniques (Durrant-Whyte and Henderson, 2016) and state estimation methods (Thrun, 2002) for integrating limited modal data, the field has evolved significantly. In contrast to these traditional single-modal or basic multimodal perception paradigms, modern embodied environments require the sophisticated collaborative integration of heterogeneous modal data, including visual, linguistic, haptic, and depth information (Ma et al., 2024). This requirement elevates the core challenges of multimodal perception to cross-modal semantic fusion, modeling complementary information, dynamic temporal alignment, and robustness under environmental disturbances and sensor uncertainties. With breakthroughs in the theory of joint representation of language, vision, and action (Ma et al., 2024), multimodal perception has emerged as a key enabling technology to achieve closed-loop collaboration between language instruction parsing, environmental state feedback, and adaptive task planning. Representative works such as LXMERT (Tan and Bansal, 2019) and MDETR (Kamath et al., 2021) establish semantic associations between modalities through cross-modal attention mechanisms, thus laying the theoretical foundation for perception-cognition coupling in embodied tasks. Beyond this, very recent work continues to push the boundaries of multimodal integration. The OmniSegmentor framework (Yin et al., 2025) demonstrates a universal pre-training paradigm across five visual modalities (RGB, depth, thermal, etc.), significantly boosting perceptual capabilities and setting new state-of-the-art records on multiple segmentation benchmarks. This highlights a trend towards more flexible and powerful general-purpose multi-modal perception backbones for embodied agents. The universal applicability in scenarios such as robotics, human-computer interaction, and semantic navigation further highlights the systemic value of this paradigm.

### 2.2 Modeling perceptual uncertainty and cross-modal alignment

Multimodal perception has increasingly become the core input component of Embodied Intelligent systems. However, modeling perceptual uncertainty and achieving cross-modal semantic alignment remain critical challenges. The theoretical challenge lies in establishing a paradigm for uncertainty modeling. Based on the sources of uncertainty, two fundamentally distinct theoretical categories can be identified. The first category stems from the inherent ambiguity, incompleteness, and environmental interference present in perceptual data itself, such as motion blur, sensor noise, and local occlusion. This type of uncertainty is referred to as intrinsic uncertainty, which possesses an irreparable nature. Its likelihood distribution must be modeled through mechanisms such as probabilistic heatmap prediction, multi-hypothesis output branches, and confidence regression (Kendall and Gal, 2017). The other category stems from insufficient prior knowledge in the model, typically manifested as a covariate shift between the training data distribution and the real-world scenario. This is referred to as cognitive uncertainty, and its modeling relies on theoretical frameworks such as Bayesian neural networks, Dropout random sampling (Gal and Ghahramani, 2016), and ensemble learning (Lakshminarayanan et al., 2017) to enhance the model’s robustness in unknown scenarios.

**FIGURE 3:**
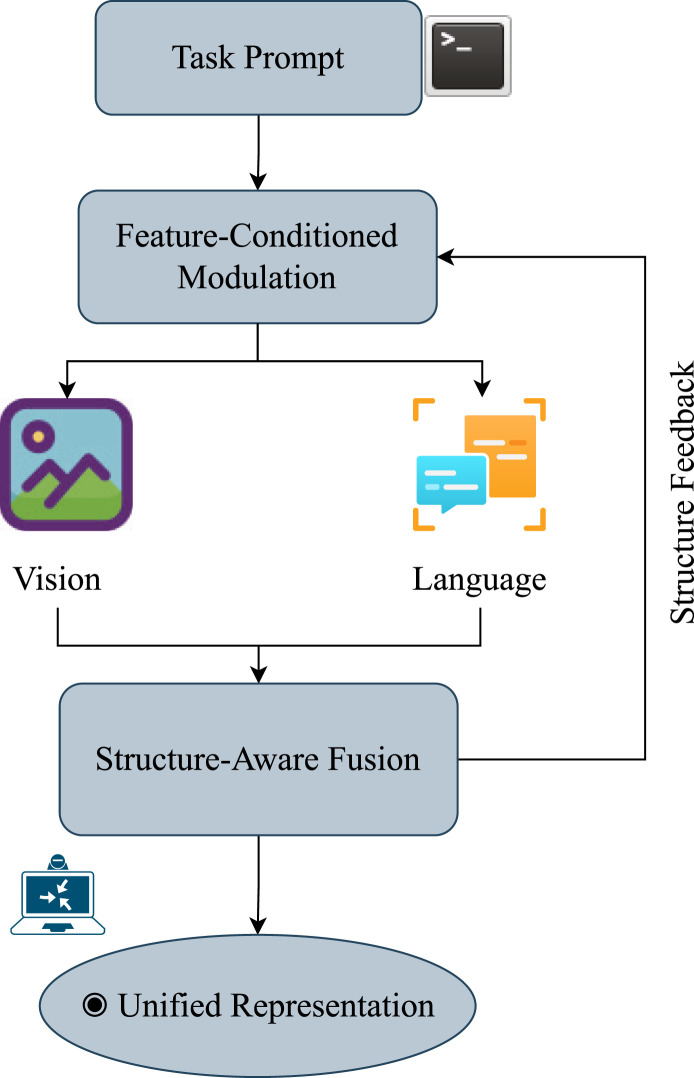
Diagram of the proposed Feature-Conditioned Modality Alignment (F-CMA) framework. Task prompts modulate vision and language features through a dynamic conditioning mechanism, followed by structure-aware fusion to ensure geometric and semantic consistency. A feedback path maintains structural alignment under uncertainty.

In embodied tasks, these two types of uncertainty often overlap and are further influenced by the heterogeneity among multimodal inputs. For instance, semantic ambiguity between vision and language modalities, as well as modality loss caused by sensor failures, can significantly interfere with policy learning and decision-making. To address this challenge, previous studies have proposed a series of cross-modal alignment mechanisms (see, e.g., (Hossain et al., 2025), Fed-CMA (Chen et al., 2020)) to mitigate the propagation of inconsistencies to downstream tasks. While these alignment methods cover a wide range of strategies, from explicit mappings (e.g., image-text labels) to implicit attention mechanisms (e.g., cross-modal Transformer fusion), they lack the ability to dynamically perceive task objectives. Consequently, when task semantics change or input modalities shift, such methods often struggle to achieve reliable alignment and adaptive fusion. For example, in language-guided grasping tasks, systems frequently cannot make robust fusion decisions when confronted with ambiguous language goals (e.g., “grab the edge”) or occluded image modalities. To mitigate these limitations, particularly the lack of semantic regulation and structural preservation during cross-modal alignment, recent work has incorporated the concept of feature-conditioned guidance into local mechanisms. For example, FiLM (Perez et al., 2022) modulates the normalization parameters of the visual branch using language features, enabling conditionally aware visual encoding; MDETR (Kamath et al., 2021) uses text fragments as queries to explicitly guide the detection and alignment of objects in images, establishing semantic correspondences between phrases and image regions; BLIP-2 (Li et al., 2023) introduces a phased freezing and multimodal alignment modulation mechanism in its training policy, achieving cross-modal semantic modeling while maintaining the stability of the unimodal encoder structure; VIMA (Jiang et al., 2022) uses language prompts as conditional inputs for policy generation, achieving a conditional bridge from perception to action. Very recently, research in 2024 has further advanced this paradigm by developing more generalizable and robust conditionally-aligned models. For instance, (Belkhale et al., 2024), introduced RT-H (Robotic Transformer with Hierarchical Planning), which leverages a language-conditioned transformer architecture to generate precise motor control commands from complex natural language instructions and visual inputs, demonstrating superior real-world performance. These latest efforts highlight a clear shift towards building large-scale, task-aware, and semantically grounded conditional alignment systems that are both scalable and adaptable. Although these methods have different application objectives, they fundamentally embody the prototype mechanism of “using explicit features from one modality as conditions to guide the representation process of another modality.” Therefore, this study unifies such mechanisms under the name “Feature-Conditional Modality Alignment (F-CMA),” as illustrated in Figure 3. This paradigm is well-suited to the need for deep collaborative modeling of heterogeneous modalities in embodied intelligence, enabling task-semantics-driven multimodal fusion and uncertainty modeling.

## 3 Policy generation and task adaptation

The problem of how an agent maps world states to actions—the policy—has deep roots in control theory and early AI. Classical approaches ranged from deliberative planners (Fikes and Nilsson, 1971) that operate on symbolic representations to reactive, behavior-based systems (Brooks, 2003) that emphasize a tight coupling between sensors and actuators. These establish the fundamental trade-offs between planning and reactivity. In contemporary Embodied AI, the policy module continues to play a pivotal role, but its challenges have expanded. Operating in dynamic environments, it must not only comprehend the current state but also generate behavioral decisions characterized by generalization capability, stability, and efficiency, all under the guidance of complex semantic task objectives.

### 3.1 Cognitive positioning of the policy module

The traditional definition of policy is limited to state-action mapping modeling. For embodied intelligence, this definition is clearly overly simplistic. Embodied systems must operate in open environments, utilizing multi-source sensory inputs, dynamic uncertainties, and diverse constraints arising from complex task objectives. Consequently, the policy module should be conceptualized not as an “end-effector controller” but as a Structural Task Adapter (STA). The role of the policy module in Embodied Intelligence should be reinterpreted as a multi-task decision path generator based on perceptual states and semantic goals, possessing task restructuring, resource scheduling, and semantic alignment capabilities. The design of the policy module should not be limited to mapping modeling but should include the following three cognitive functions. First, the policy should internalize the ability to model semantic task conditions. It should not merely “see the state and perform actions” but should deeply understand “what my current goal is” and seamlessly integrate task conditions in the form of text instructions or semantic graphs into the policy generation process. For example, text instructions are converted into embeddings and directly drive a multimodal Transformer for control prediction in the RT-H (Belkhale et al., 2024) system; and Prompt-DT (Xu et al., 2022) encodes instructions and environmental trajectories together into generated plan tokens, which are typical examples of this capability. Second, the policy must have the core functionality of structured path generation. Modern embodied policies no longer output a single action but instead output structured trajectories that include causal dependencies between sub-goals. For example, DreamerV3 (Hafner et al., 2025) performs “inner loop prediction” in the world model to generate plans; SayCan (Ahn et al., 2022) uses an LLM to output multi-step sub-tasks, which are then executed by the actuator. Therefore, the policy network should have temporal awareness, semantic consistency, and planning capabilities (Kaelbling and Lozano-Pérez, 2011). Finally, the policy must achieve task scheduling and system interface decoupling, with the policy module automatically selecting sub-policies, functional modules, or control parameters based on task conditions. Successful implementations in multi-task systems like Gato (Reed et al., 2022) and ChatVLA (Zhou et al., 2025) demonstrate that policies are no longer “functions” but flexible “scheduling entry points.”

Within the DP-TA theoretical framework, the policy generation and adaptation layer occupies the top-level decision-making module of the system. Its inputs integrate multi-modal environmental states provided by the perception and alignment layer (Parisi et al., 2022), future states predicted by the world modeling layer and causal inference chains (Kaelbling and Lozano-Pérez, 2011), as well as semantic goal streams defined by user tasks (Ahn et al., 2022) or LLM (Xie et al., 2023) modules. The outputs of the policy layer must satisfy three key requirements: 1) direct executability by controllers or downstream modules (Liang et al., 2022); 2) effective responsiveness to task variations and resource constraints (Ge et al., 2023); 3) interpretability through visualization and analysis in the form of tokens, trajectories, or graph formats. The hierarchical structure of DP-TA relieves the policy module of the entire inference burden, freeing it to focus on integrating contextual structure and semantic goals; thereby, it functions as an efficient task scheduler (Liang et al., 2022), representing a fundamental departure from traditional pipeline-based control systems. In summary, the cognitive positioning of embodied policy modules is evolving from “action selectors” to “structural task schedulers” to support multimodal semantic fusion, task decomposition, and dynamic module invocation (Liang et al., 2022; Brohan et al., 2022).

### 3.2 The evolutionary path of policy mechanisms and prompts

The policy generation mechanism in embodied intelligence has evolved progressively from action-supervised learning to structured decision generation. This evolution signifies a paradigm shift from “input-dimension constraints” to “structure-modeling enhancement” (Levine et al., 2020), underscoring the transformation of policy modules from purely executive functions toward cognitive systems. Contemporary mainstream approaches to policy generation can be broadly classified into three systematic paradigms: mapping-based, optimization-based, and structure-based.

The mapping paradigm represents the most direct supervised learning path for early perception-action systems, with Behavior Cloning (BC) (Torabi et al., 2018) and Imitation Learning (IL) (Zheng et al., 2024) serving as its representative methods. Its core assumption is that, given a state, supervised learning can approximate the expert policy, thereby achieving an end-to-end mapping from perception to action. Methods within this paradigm, exemplified by systems such as RT-1 (Brohan et al., 2022) and BC-Z (Jang et al., 2022), demonstrate effective cold-start performance, particularly in fixed-process, single-task operational workflows. However, this modeling approach treats task objectives as implicit conditions, resulting in a policy that lacks adaptability to instruction variations or environmental dynamics (Shafiullah et al., 2022). Consequently, when task objectives change, the policy cannot structurally adapt, leading to performance degradation in multi-task or temporally extended planning scenarios. Although characterized by high training efficiency, the inherent lack of structured representation within the policy hinders support for intra-task causal reasoning or cross-semantic transfer.

The optimization paradigm is based on reinforcement learning, which uses reward-driven behavioral strategy learning as its theoretical foundation. Starting from the task objective, the policy is iteratively optimized through reward signals (Josic, 2021). Modeling typically combines a policy function with a state-value function or Q-function. In embodied tasks, systems like DreamerV3 (Hafner et al., 2025) leverage world models for “virtual interaction,” significantly enhancing learning efficiency and environmental generalization capabilities (Schrittwieser et al., 2020). This approach incorporates long-term reward modeling and emergent policy self-organization, making it theoretically more suitable for complex goal-oriented planning. Nevertheless, it faces practical challenges including training instability and high sample complexity (Dulac-Arnold et al., 2019). Furthermore, the tight coupling between the policy and its training environment compromises module transferability and policy reusability.

Recent research is advancing strategic modeling towards a structural planning paradigm. Here, language prompts, planning structures, and causal graphs serve as the primary entry points for strategy control. Prompts have evolved beyond mere instructions to function as task-driven mechanisms for strategy configuration (Shin et al., 2020). At the foundational level, task conditioning provides the core task semantics (“what to do”), exemplified by directives such as “place the red cup on the left shelf”. At a deeper level, behavioral guidance navigates complex strategy spaces by offering “how to do it” directions. This effectively creates a “semantic roadmap” that identifies the optimal behavioral choice within multi-strategy contexts (Belkhale et al., 2024). The highest level structural interface transforms natural language or semantic prompts into tokens, graphs, or intermediate representations. This facilitates the strategy module’s uniform processing of interface information spanning language, perception, and control. This methodology departs from conventional approaches that directly learn state-to-action mappings. Instead, it frames strategy modeling as a structured generation task: sub-goal sequences, planning tokens, or semantic graphs are first constructed and subsequently decoded into specific control instructions.

Integrating Prompts Deeply into Control Pipelines. The SayCan system exemplifies the “language prompt → sub-goal decomposition” approach. In this paradigm (Ahn et al., 2022), user instructions are initially processed by a large language model (LLM) to generate a structured sequence of sub-tasks (e.g., pick → move → place). Subsequently, an actuator selects executable modules based on the current perceptual state, while a dedicated strategy controller handles only the final execution step. This approach significantly enhances strategy interpretability and composability. However, its effectiveness is critically dependent on the planning quality of the external LLM, and it lacks a real-time feedback loop during execution. Conversely, the Prompt-DT paradigm employs a “prompt token → action sequence generation” strategy. Here, natural language prompts are concatenated with state trajectories and fed into a Transformer decoder to predict sequences of future behavior tokens (Xu et al., 2022). This method effectively integrates the efficiency of imitation learning with the flexibility of prompts, demonstrating robust multi-task adaptability and stable performance under low-data regimes. Its key innovation lies in decoupling task abstraction from the training architecture, thereby supporting instruction-level nesting and generalization. Representing the most promising end-to-end solution, the RT-2 (Zitkovich et al., 2023) system embodies the “unified token representation → multimodal policy control” paradigm. It encodes heterogeneous inputs (e.g., images, language instructions) into a unified token sequence, which is then processed by a multi-layer Transformer for direct strategy prediction and control output. This architecture not only unifies the input processing pipeline but also establishes a genuine end-to-end closed loop bridging perception and control. Such advancements are poised to endow strategy modules with “elastic structures” and “adaptive computation paths,” ultimately realizing a unified decision-making loop that translates natural language instructions into structured, interpretable, and high-performance behavioral responses.

### 3.3 System adaptation of the policy module

Embodied Intelligent systems are increasingly deployed not in closed experimental settings, but in open, dynamic, and resource-limited real-world environments. Consequently, the evaluation metrics for strategy modules extend beyond mere “task completion rate” or “reward function maximization” to encompass.

**FIGURE 4:**
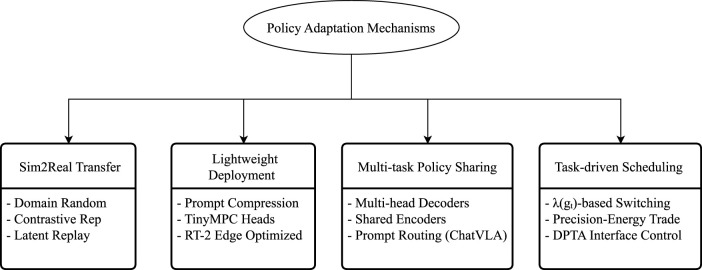
Four key pathways for policy adaptation in embodied systems: Sim-to-Real transfer, lightweight deployment, multi-policy sharing, and task-aware precision scheduling.

A comprehensive set of factors includes system load capacity (latency, power consumption); multi-task versatility (prompt support, semantic compatibility); training-to-deployment transferability (Sim-to-Real stability (Liu et al., 2024), module consistency); and structural compression capability (decoder depth, module reusability). This paradigm shift necessitates the evolution of the strategy module beyond its traditional “strategy modeling” function into a “System Adapter.” This adapter forms a mediating layer between the strategy structure and deployment logic, implementing four key adaptation pathways (as illustrated in Figure 4). Figure 4 summarizes the four primary adaptation mechanisms employed in the strategy module design of current mainstream embodied systems, spanning critical areas such as simulation transfer, lightweight deployment (Xiao et al., 2023), task offloading, and control precision scheduling.

First, the simulation-reality gap remains a core challenge in deploying embodied control systems. Relying solely on the policy itself is insufficient to address domain shifts, necessitating auxiliary mechanisms to ensure consistency in state representations. For instance, DreamerV3 (Hafner et al., 2025) enhances policy stability significantly by combining world model-based recurrent rollouts with dynamic reward reconstruction. Similarly, Prompt-DT (Xu et al., 2022) leverages language prompts to guide trajectory generation, maintaining alignment between instructions and behaviors across diverse task objectives, thereby demonstrating strong real-world transferability. Ultimately, Sim-to-Real is not a perception issue but fundamentally a challenge of structural consistency, requiring the establishment of a structural identity mapping between policy inputs, state representations, and trajectory generation logic.

Second, lightweight policy deployment is critical, particularly in edge devices or multi-task systems where model size and inference latency become key bottlenecks. Current mainstream approaches include utilizing the Prompt Pool mechanism to compress multiple instructions into a shared token space; replacing the policy decoder with structures like TinyMPC or LightweightHead; and strategically reducing input resolution while enhancing instruction semantic quality to achieve a complementary “control precision-input intensity” trade-off. Beyond these algorithmic optimizations, groundbreaking work in full-system co-design has demonstrated the feasibility of deploying complex policies on affordable, mobile platforms. The Mobile ALOHA 2 system (Aldaco et al., 2024) exemplifies this by integrating a compact bimanual hardware design with a tailored imitation learning pipeline, enabling low-cost mobile manipulators to execute sophisticated bimanual tasks with high precision and robustness. This approach highlights that effective lightweight deployment often requires joint innovation across policy algorithms, hardware design, and data infrastructure. Taking RT-2 as an example, its policy network leverages multimodal embedding and hierarchical token fusion to maintain inference latency below 150 ms at 92 % accuracy (Zitkovich et al., 2023), demonstrating practical deployability.

Third, multi-policy shared architectures offer a cost-effective solution for embodied systems, which typically encounter evolving tasks rather than structural changes. Consequently, a single universal policy is far less efficient than a shared backbone structure coupled with a dynamic switching mechanism. In the ChatVLA framework (Zhou et al., 2025), language prompts are employed to select sub-policy paths, while all tasks share the main representation module (visual → semantic → control). This mechanism achieves lightweight module invocation through structural distillation and adapter injection, maintaining <5 % accuracy degradation across nine distinct tasks while reducing memory usage by over 60 % .

Fourth, task-aware precision adjustment is essential for embodied systems operating continuously or under power constraints, requiring policy modules to be resource-sensitive to task objectives. This can include reducing decoder depth, activating lightweight execution branches, or integrating external planners, for example, leveraging a world model to preprocess predictions. Within the DP-TA architecture, the policy layer can serve as a precision-aware routing module, enabling resource-efficient control while maintaining task effectiveness.

### 3.4 Unified interface and pathway design for policy modules in the DP-TA architecture

Within traditional embodied system architectures, the policy module is typically treated as the “model tail” or a “control module.” Its inputs and outputs are often tightly coupled with the specific implementation of preceding network components, lacking inherent structural independence and a well-defined interface. To address this limitation, we advocate for a redefinition of the policy layer within the DP-TA framework as a structurally explicit, interface-unified, and semantically autonomous module. This module bridges perception-modeling outputs with downstream execution demands, adapting flexibly to varying task objectives. From a structural perspective, the DP-TA’s policy generation and adaptation layer receives two types of information: one from the perception and alignment layer’s modal fusion state and the other from the world modeling layer’s dynamic state prediction. These inputs, integrated with task-specific objectives such as language prompts, graph structures, and skill descriptions, constitute the complete input set for the policy module. Its outputs encompass three critical elements: continuous motion commands (e.g., 6D poses (Wen et al., 2024) or trajectory point sequences (Bui et al., 2020)) directed to the robot controller; behavior routing directives (e.g., sub-policy IDs or skill invocation signals) governing mid-level skill orchestration; and structured plan graphs or trajectory confidence estimates provided to the explanation layer, furnishing verifiable decision evidence for human oversight.

**FIGURE 5:**
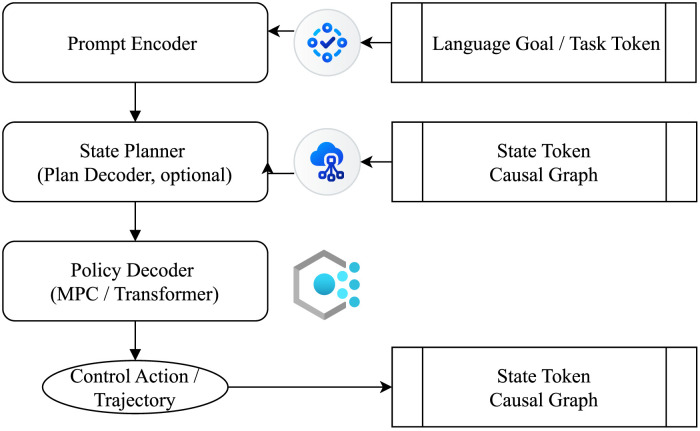
Unified structure and interface pathways of the policy module in the DP-TA architecture.

The core contribution of the DP-TA policy layer is not simply to identify “which policy performs best” but to define a unified structural interface that accommodates a diverse ensemble of policy mechanisms—including behavior cloning, reinforcement learning, and model predictive control. This design enables the integration of a policy library (policy ensemble) with a routing mechanism (policy router) (Lou et al., 2023), facilitating the dynamic selection, reconfiguration, and composition of policies based on contextual needs. Rather than focusing solely on selecting the best-performing policy model, we argue that a more critical research direction is how to architect a controllable and configurable policy space—one that can generalize across tasks, adapt across platforms, and respond to heterogeneous input modalities. To concretize this abstraction, Figure 5 illustrates the modular composition, interface pathways, and functional decoupling within the DP-TA policy generation layer.

In summary, the role of the policy module in DP-TA extends far beyond single action generation. It provides structured interfaces, enables semantics-driven scheduling, and facilitates controllable policy generation for complex systems. This conceptual shift establishes a novel paradigm for future embodied intelligence research, redirecting focus from individual model performance towards the structural design of the policy space and the modeling of execution capabilities. This approach paves the way for achieving genuine cognitive unification and task generalization.

## 4 The structural function and system integration of world models in embodied intelligence

### 4.1 The evolutionary trajectory of model structure

The concept of an internal world model—an internal representation that allows an agent to simulate and predict the consequences of its actions—is a foundational idea in cognitive science and AI. It was central to the “sense-model-plan-act” paradigm (Nilsson, 1984) that dominated early robotics. While limited by computational power, these early models, often based on Bayesian filters (Kalman, 1960) or graphical models, aimed to maintain a belief state about the world.

Today, the term “World Model” (WM) has been revitalized by deep learning. Modern WMs, powered by vast data and neural networks, comprehend the dynamics of the real world at an unprecedented scale, encompassing its physical and spatial properties. Utilizing multimodal input data including text, images, videos, and motion, they generate predictive video sequences (Ding et al., 2025). Through learning, these models acquire an understanding of the physical characteristics governing real-world environments, enabling them to represent and forecast dynamics such as motion, stress, and spatial relationships within sensory data. Within the progression of embodied intelligence, the architectural evolution of world models has advanced from initial RNN encoders (Yu et al., 2022) through latent state space modeling to contemporary multimodal Transformers endowed with structural awareness and causal reasoning capabilities (Zhang et al., 2023). This evolution is now accelerating with the rise of generative world models that learn controllable dynamics from internet videos. Notable examples include Genie (Bruce et al., 2024), which generates actionable 2D worlds from image prompts, and its successors that explore 3D physical reasoning (Shang et al., 2025).

This trajectory reflects three key evolutionary trends: from perceptual representation to state latent variable abstraction; from single-step prediction to trajectory rolling simulation and reward estimation; and from continuous variable generation to structural token combination modeling. A further significant evolution involves the shift from 2D-centric to 3D-grounded representations and from discriminative to generative world modeling. The recently proposed 3D-VLA model (Zhen et al., 2024) epitomizes this trend. It is built upon a 3D-based large language model (LLM) and introduces a generative world model that predicts future 3D scenarios (as point clouds and images) through embodied diffusion models. This approach moves beyond direct perception-action mapping by enabling the model to ‘imagine’ the consequences of actions in a 3D space prior to planning, thereby seamlessly integrating 3D perception, reasoning, and action generation. These trends signify that world models are no longer merely auxiliary tools for policy training but are increasingly becoming structural modules with independent cognitive functions within embodied intelligence systems.

**FIGURE 6:**
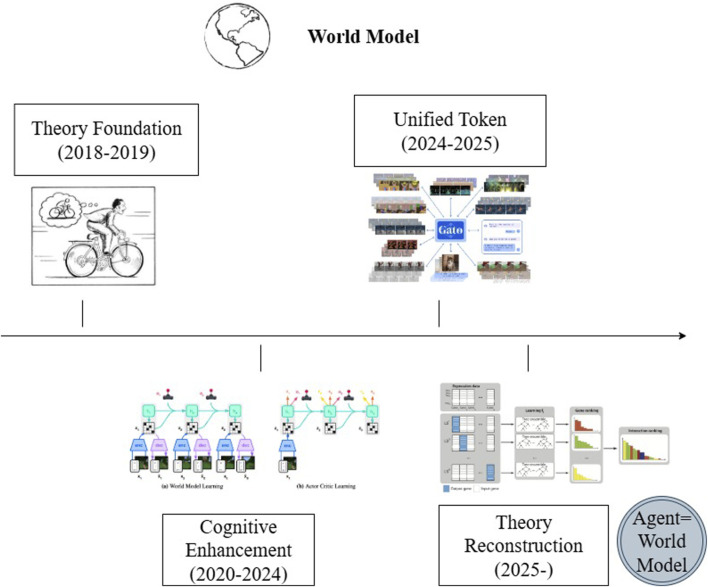
It illustrates the development trajectory from world models to representative systems such as DreamerV3 (Hafner et al., 2025), Gato (Reed et al., 2022), and DeepMind Genie. Each stage in the diagram summarizes its technical features and key models in chronological order, reflecting the evolution of world models from predictive tools to the core of cognitive structures.

As illustrated in Figure 6, contemporary world model designs are expanding from modeling mere state transitions to generating semantic structural graphs. This expansion provides enhanced interpretability and multi-task adaptability for policy generation. The underlying model structures now seek a new equilibrium between abstract representational unification and explicit structural interpretability. Modern research on world models is thus undergoing a conceptual transition—from optimizing for predictive fidelity to emphasizing structural coherence and causal planning capability. This evolution positions the world model not merely as a support module for behavior, but increasingly as a central inferential agent within embodied cognitive systems. In conclusion, the ultimate goal of world modeling is not simply to replicate the external world, but to construct a structural proxy—a model capable of generating semantic graph representations that can inform strategy synthesis, instruction interpretation, and system-level coordination. This redefinition elevates world models to the role of cognitive infrastructure, essential for achieving generalizable and interpretable embodied intelligence.

### 4.2 The three functions of world modeling

Within embodied intelligent systems, the role of world models extends far beyond functioning as mere environment simulators or state predictors (Ha and Schmidhuber, 2018b). We argue that world models should be redefined as structural state inference engines within the system. Their crucial responsibilities extend beyond dynamic modeling to enabling the system to achieve a functional closed-loop encompassing structural perception, cognitive simulation, and task planning across three key dimensions. First, State Reconstruction addresses the spatio-temporal alignment issues of heterogeneous modalities such as vision, language, and haptics. Distinct from the instantaneous representations generated at the perceptual layer, world models focus on capturing the dynamical concepts inherent in historical state sequences. A representative example is the Recursive State Space Model (RSSM) (Hafner et al., 2019b) utilized in DreamerV3 (Hafner et al., 2025). This mechanism encodes latent states to capture the structural evolution of the environment, thereby establishing a structured representational foundation for subsequent reasoning. Next, Behavior Simulation builds a virtual interaction space based on the representation, evaluating the long-term benefits of action sequences through rollout prediction (e.g., MPC optimization), significantly reducing the cost of real-world interaction. The frontier of this research is moving towards highly controllable and fine-grained simulations. The GEM model (Hassan et al., 2025) exemplifies this direction: it is a generative world model that predicts future ego-vision frames with precise, independent control over object dynamics, ego-agent motion, and human poses by conditioning on sparse features, trajectories, and pose data. By generating paired RGB and depth outputs autoregressively, GEM enables a rich, spatially-aware simulation space for testing diverse ‘what-if’ scenarios, greatly enhancing the robustness and versatility of policy learning in complex, multi-agent environments. Finally, Causal Graph Induction (CGI) essentially establishes an explicit reasoning chain of “goal → action → outcome”. This form of structured causal reasoning not only facilitates explainable task decomposition but also endows the system with counterfactual reasoning capabilities—predicting the potential consequences of actions not taken, which is critical for safety-aware decision-making in high-risk scenarios.

### 4.3 Cognitive architecture for world modeling and strategy coordination

In an ideal embodied intelligent system, the world model and the policy module should form a stable cognition–control feedback loop. Specifically, the world model handles structural state modeling and causal planning, while the policy module determines behavioral trajectories based on the world model’s outputs (Rohekar et al., 2024). This interaction forms a closed-loop information flow integrating environmental perception, internal modeling, and execution control. Such coupling is reflected not only in the data flow but also in the functional alignment and structural co-design of the two modules. The following discusses three mainstream approaches to achieving this integration.

The most basic form of coordination treats the world model as a state prior generator for the policy. For instance, Dreamer (Hafner et al., 2019a) leverages a learned environmental dynamics model to generate synthetic trajectory rollouts. Concurrently, an internal reward model optimizes policy parameters. In this setup, the task of policy training is effectively delegated to the simulated environment, decoupling learning from physical interactions and significantly improving cross-environment generalization.

A more structured approach organizes the world model and policy module into a hierarchical decision-making pipeline. Here, the world model functions as a structural planner, decomposing high-level goals into structured sequences of sub-tasks (e.g., “clean spilled liquid on the table → fetch cloth → wipe surface”), while the policy module selects low-level control strategies under given constraints to execute each step. This architecture offers a clear separation between planning and execution, well-defined module boundaries, and strong interpretability. However, it places stringent demands on logical consistency—inconsistent sub-goals (e.g., “move an unsecured object”) may lead to irrecoverable execution failures.

The most advanced trend adopts a unified token-based architecture, embedding both the world model and policy module within a single Transformer framework to enable token-level inference–execution coupling. In this design, state observations, language instructions, and behavioral tokens are embedded in a single input sequence. Causal attention mechanisms are then employed to model cross-modal temporal dependencies (Chen et al., 2021). The policy decoder subsequently outputs either the next action or module invocation within the same token stream. This unified approach, exemplified by systems such as Gato and RT-2, tightly integrates perception → prediction → decision into a shared latent space, achieving minimal architectural complexity, high coupling strength, and fast response to instructions (Reed et al., 2022; Zitkovich et al., 2023). Within the DP-TA framework, this paradigm reflects a convergence of structural compression and real-time scheduling.

### 4.4 Interface paths and functional coupling of world models in DP-TA structures

Within the three-layer DP-TA architecture, the world model constitutes the intermediate modeling layer, functioning as a cognitive mediator that bridges perception outcomes and policy control. Unlike conventional approaches that regard the modeling module as a loosely connected “task simulator” or “auxiliary predictor” (Ha and Schmidhuber, 2018a), we advocate for a more central role: the world model should be viewed as a generator of structured states, a simulator of behavioral trajectories, and the inference backbone for task graph construction. As the modeling core of the DP-TA architecture, the world model not only enables seamless information flow between perception and control but also provides a unified structural foundation for multi-task adaptation and reasoning-based execution.

## 5 DP-TA theoretical structure

The design of contemporary embodied intelligence systems typically adheres to a modular paradigm, decomposing complex systems into relatively independent submodules—such as perception, planning, control, and world modeling. This modular approach builds upon classical robotic architectures that date back to the early sense-plan-act paradigm. While this design facilitates independent development, optimization, and iteration of each component, it often fails to explain how the system as a whole can adapt to dynamic tasks, coordinate resource allocation, and execute coherent behaviors (Batra et al., 2020). In recent years, multimodal large model (MLM)-based embodied systems, such as those leveraging LLaVA and Gemini, have emerged as a dominant direction (Liu et al., 2023; Team et al., 2023). These systems inherently challenge traditional modular boundaries, favoring unified architectures with joint perception, reasoning, and action generation. Parallel efforts (e.g., Meta’s Habitat 3.0 and Stanford’s Mobile ALOHA) focus on zero-shot task generalization in open and unstructured environments (Puig et al., 2023; Fu et al., 2024). Yet, generalization to novel objects remains a critical gap in current research. To address this limitation, we integrate classical principles with modern advances in DP-TA (Dynamic Perception–Task Adaptation) as a response to the structural fragmentation of existing embodied systems. DP-TA aims to enhance generalization to unknown objects and to explore a unified theoretical framework that integrates system functionality across hierarchical levels and task workflows. It provides a conceptual foundation for encapsulating perception inputs, world modeling, and policy control into standardized, composable, and schedulable task pipelines, facilitating greater adaptability and coordination in real-world, dynamic environments.

### 5.1 Three-layer functional structure of the DP-TA framework

The DP-TA framework builds upon the classical perception-modeling-control paradigm by introducing a three-layer system architecture. Each layer independently encapsulates a core system function, while structurally forming a closed loop via an intermediate state interface and a semantic token alignment mechanism. The Perception and Alignment Layer (P-layer) integrates multi-modal sensory inputs (e.g., vision, haptics) to produce semantically aligned token sequences that serve as unified state representations for downstream modeling and control modules. The World Modeling and Structure Prediction Layer (W-layer) simulates environmental dynamics and predicts task-relevant structures to generate executable plans and action paths. The Strategy Generation and Adaptation Layer (T-layer) formulates concrete action strategies based on task goals and environmental states, while dynamically adjusting execution in response to environmental feedback. Although functionally distinct, these three layers are structurally interconnected through standardized interfaces and a shared semantic token space, forming a theoretically closed-loop system. This architecture extends traditional layered approaches by providing explicit coordination mechanisms, enabling more flexible task adaptation and enhanced generalization capabilities.

**FIGURE 7:**
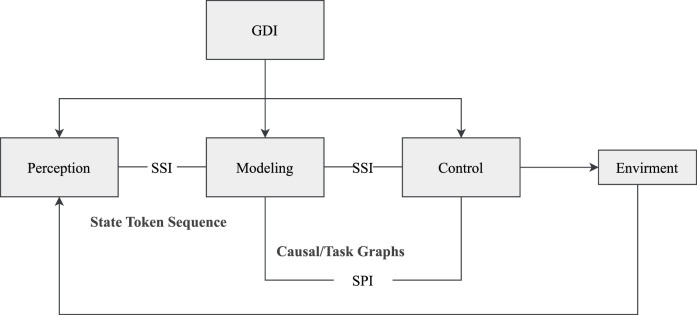
Layer-3 interface coordination mechanism.

To facilitate effective coordination among the layers during runtime, the DP-TA framework defines three standardized interfaces: the Semantic State Interface (SSI), the Structure Planning Interface (SPI), and the Goal Dispatch Interface (GDI). The SSI standardizes the output of state token sequences, serving as a task-oriented communication protocol between perception, modeling, and control modules. Inspired by hierarchical state representation methods found in classical world modeling frameworks such as Dreamer, the SSI ensures the semantic consistency of multimodal information across all layers. The SPI delivers structured outputs from the modeling layer in the form of interpretable causal graphs, task graphs, or sub-task dependency trees, enabling the strategy layer to understand and act upon the underlying task logic. The GDI enables top-down feedback from the strategy layer to the perception layer. Driven by task objectives or external prompts, it dynamically modulates perception routing, modeling resolution, and strategic planning pathways, as illustrated in Figure 7.

### 5.2 System coordination mechanism

To ensure theoretical coherence throughout the execution of the DP-TA three-layer architecture, the framework emphasizes coordination in data flow, functional roles, and structural adaptation. By transmitting state representations, predicted states, and behavior tokens across the three layers, DP-TA enables continuous and closed-loop information processing, ensuring both fluidity and consistency of data. The perception layer is responsible for generating semantic representations from multimodal sensory data; the modeling layer simulates environmental dynamics and predicts future states; and the policy layer formulates executable action plans. These layers form a progressive processing pipeline from perceptual input to action output. To facilitate interoperability, modules across all layers are designed to support a unified embedding format (e.g., token-based nested representations), shared attention mechanisms, and standardized interfaces for planning graphs. Such structural adaptation mechanisms enhance inter-module compatibility, system scalability, and cross-modal integration. The DP-TA framework draws inspiration from the form–behavior–learning paradigm, positioning state evolution as a central link that bridges semantic perception and action generation. For example, in a robotic manipulation task such as grasping a green object on a table, the DP-TA framework enables a closed-loop process from natural language instruction to physical execution. The perception layer detects the green object through visual and tactile sensing, generating a coherent state representation. The modeling layer predicts environmental dynamics and plans the grasping trajectory. The strategy layer then produces an object-specific grasping plan, dynamically adapted via a Grasp-Driven Interaction (GDI) mechanism. This enables the robot to determine how to grasp the object based on its unique properties and context. This closed-loop pipeline showcases the potential of DP-TA for tackling complex, multimodal tasks in embodied intelligence systems.

The DP-TA framework thus represents an evolution rather than a revolution—it systematizes and extends classical robotic principles with modern neural architectures, offering a unified theoretical approach to addressing the issue of modular fragmentation in embodied intelligent systems. By establishing a three-layer functional architecture, standardized interfaces, and collaborative coordination mechanisms, DP-TA creates a structured collaboration pathway for task-level integration, thereby providing theoretical guidance for system-level design. Its strengths in multi-agent collaboration, multi-modal integration, and interpretability position DP-TA as a promising research direction within the field of embodied intelligence. While its practical deployment still requires further investigation, the framework offers a coherent theoretical foundation and a standardized implementation pathway for the development of next-generation intelligent systems.

## 6 Research challenges

Although embodied intelligent systems have made substantial progress in perception modeling, language-conditioned control, world modeling, and cross-modal alignment, achieving system architectures that are structurally generalizable, semantically interpretable, and deployable in a controllable manner remains a significant challenge.

First, at the perception and alignment layer, semantic mismatches across modalities are still prevalent. Even with the application of large-scale models for joint image–language modeling, modal output inconsistencies persist in complex environments characterized by occlusion, weakened sensory representations, or ambiguous task objectives (Liu et al., 2025). These stem primarily from the lack of task-conditioned cross-modal consistency constraints. Future directions may include the development of task-aware cross-modal routing mechanisms to adaptively align modalities under varying task demands. Moreover, language prompts are still difficult to translate directly into structured state representations. Current perception modules lack the capacity to convert high-level linguistic task instructions into attention-guided structures in state space, limiting their effectiveness in task transfer and generalization.

Second, at the world modeling layer, current models struggle with structural task graph construction. Most world models remain confined to short-term predictive tasks, lacking the capability to explicitly model complex instruction hierarchies and causal chains of subtasks. A promising direction is the development of a language–behavior–causality Transformer that enables multidimensional joint modeling (Ding et al., 2025). In addition, long-horizon prediction models often suffer from high training costs and poor generalization. Training a stable world model requires extensive rollouts and high-dimensional reconstructions, but the resulting models are often vulnerable to behavioral drift and offer limited trajectory control in unseen environments.

**FIGURE 8:**
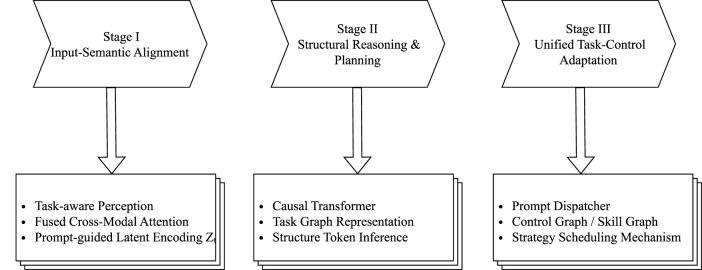
The three-stage evolution pathway of embodied intelligence systems. This pathway illustrates the progressive integration from multimodal perception to adaptive action generation.

Third, at the strategy generation layer, the formulation of strategy paths is still predominantly dictated by predefined model architectures. Most current multi-task policy systems rely on hard-coded modules or fixed policy heads (e.g., multi-head architectures), rather than being dynamically driven by semantic task goals. This reveals a lack of intermediate structural representations that bridge task semantics and control strategies. Furthermore, strategy–resource coupling mechanisms remain underdeveloped. Critical components such as policy switching, precision adaptation, and energy consumption control have yet to be seamlessly integrated into multi-task systems, resulting in challenges in system stability, scalability, and maintainability during deployment. The three-stage evolution path of embodied intelligent systems from perception alignment to structural modeling and then to strategy control as illustrated in Figure 8.

## 7 Summary and prospects

The development of embodied intelligence has evolved beyond isolated perception and control tasks into a systematic research paradigm that encompasses three fundamental pathways: perceptual input, world state modeling, and behavior generation. However, current research efforts often remain focused at the modular optimization level, lacking unified structural standards and task-driven architectural guidance. This phenomenon—characterized by local refinement but systemic fragmentation—significantly hinders the transferability, semantic adaptability, and practical deployability of embodied systems. This study conducts a comprehensive analysis of this *status quo*. First, we review recent advancements in multimodal perception and dynamic alignment, and synthesize existing work to propose the concept of Feature-Conditioned Modal Alignment (F-CMA). Next, we examine the role of the strategy module in embodied systems and investigate the evolving functions of world models. We argue that a stable cognition–control feedback loop should be established between the world model and the strategy module to support adaptive, task-aware decision making. To address the aforementioned challenges, we propose the DP-TA three-layer structural framework as a principled architectural solution. By decomposing the system into three functionally autonomous, interface-standardized, and semantically closed-loop layers, namely, perception alignment, world modeling, and strategy generation, DP-TA offers not only a reference paradigm for assessing the structural completeness of embodied intelligence systems, but also a clear roadmap for advancing system integration and inter-module collaboration. We hope that the structural-cognitive perspective and system integration framework proposed in this review will serve as a conceptual foundation and shared design language for future research in the field of embodied intelligence.
